# Metabolomic and Lipidomic Analysis of the Heart of Peroxisome Proliferator-Activated Receptor-γ Coactivator 1-β Knock Out Mice on a High Fat Diet

**DOI:** 10.3390/metabo2020366

**Published:** 2012-06-01

**Authors:** Gregor McCombie, Gema Medina-Gomez, Christopher J Lelliott, Antonio Vidal-Puig, Julian L Griffin

**Affiliations:** 1 Department of Biochemistry; 80 Tennis Court Road, University of Cambridge, Cambridge, CB2 1GA, UK.; 2 Metabolic Research Laboratories, University of Cambridge, Level 4, Institute of Metabolic Science, Addenbrooke’s Hospital, Cambridge, CB2 0QQ, UK.; 3 Department of Biosciences, CV/GI iMED, AstraZeneca R&D, Mölndal, S-43183, Sweden.; 4 Medical Research Council Human Nutrition Research, Elsie Widdowson Laboratory, 120 Fulbourn Road, Cambridge, CB1 9NL, UK.

**Keywords:** metabolic syndrome, obesity, peroxisome proliferator activated receptors, Peroxisome proliferator-activated receptor-γ coactivator 1-beta, functional genomics

## Abstract

The peroxisome proliferator-activated receptor-γ coactivators (PGC-1) are transcriptional coactivators with an important role in mitochondrial biogenesis and regulation of genes involved in the electron transport chain and oxidative phosphorylation in oxidative tissues including cardiac tissue. These coactivators are thought to play a key role in the development of obesity, type 2 diabetes and the metabolic syndrome. In this study we have used a combined metabolomic and lipidomic analysis of cardiac tissue from the PGC-1β null mouse to examine the effects of a high fat diet on this organ. Multivariate statistics readily separated tissue from PGC-1β null mice from their wild type controls either in gender specific models or in combined datasets. This was associated with an increase in creatine and a decrease in taurine in the null mouse, and an increase in myristic acid and a reduction in long chain polyunsaturated fatty acids for both genders. The most profound changes were detected by liquid chromatography mass spectrometry analysis of intact lipids with the tissue from the null mouse having a profound increase in a number of triglycerides. The metabolomic and lipodomic changes indicate PGC-1β has a profound influence on cardiac metabolism.

## 1. Introduction

The Western diet, consisting of a high proportion of saturated fats, is a known risk factor in a number of pathologies associated with over nutrition including type 2 diabetes, cardiovascular disease and dyslipidaemia. In addition to cardiovascular disease, cardiac metabolism is also altered in type 2 diabetes with a number of previous studies indicating that an energetic deficiency precedes the development of abnormal cardiac function in both man and mouse [1,2,3,4], although the exact mechanisms underlying this diabetic cardiomyopathy are unknown. 

In the diabetic heart, glucose consumption is significantly reduced in favor of increased β-oxidation of fats [5]. This is caused by reduced insulin-induced recruitment of GLUT4 glucose transporter to the cell membrane, decreased expression of GLUT1 and GLUT4, a decrease in pyruvate dehydrogenase activity associated with increased fatty acid oxidation and increased activation of peroxisome proliferator-activated receptor α (PPAR-α), which in turn robustly upregulates a number of genes involved in fatty acid metabolism [1,5,6,7]. All these factors produce an accumulation of glycolytic metabolites (e.g. lactate, hexosamines) and ceramides, the accumulation of the latter class of compounds being associated with increased insulin resistance in tissues [8]. Fatty acid oxidation has an increased oxygen demand compared with glucose oxidation, partly due to increased mitochondrial uncoupling [9] as well as the more reduced state of fatty acids compared with glucose. Fatty acid oxidation is also associated with increased production of reactive oxygen species (ROS) and lipotoxic intermediates such as long chain acyl carnitine derivatives in liver [10]. All of these mechanisms could ultimately result in impairment of cardiac energetics and function. However, Muoio and Newgard [10] provide compelling evidence that mechanisms defined in liver do not extend to other tissues. Indeed, they suggest that pharmacological agents that improve insulin sensitivity by increasing β-oxidation act in the liver, and do not improve muscle insulin sensitivity, and may actually be detrimental [11].

The PPARs are three nuclear hormone receptors which regulate systemic metabolism at the whole organism level in mammals, and act as nutritional sensors by coordinating metabolism across multiple organs. In addition PPAR gamma coactivator 1-alpha (PGC-1α) and PGC-1β are transcriptional coactivators which have profound influences on the PPAR system, increasing the activities of all three PPAR receptors under different circumstances. PGC-1α was originally described in brown adipose tissue as an important factor in the upregulation of thermogenesis during cold exposure [12], and both co-activators are important for the biogenesis of mitochondria [13] and the upregulation of β-oxidation in adipose, skeletal muscle and the heart [13,14]. Furthermore, PGC-1β also regulates in part the expression of carnitine palmitoyltransferase I [15], a key enzyme in the transport of fatty acids across the inner mitochondrial membrane. Both genetically induced diabetes (the ob/ob mouse) and dietary induced diabetes (high fat feeding) reduce the expression of both co-activators, with this being mediated by saturated fats [16]. Furthermore, the PGC-1 co-activators have also been implemented in the action of rosiglitazone, a potent PPAR-γ agonist used to treat type 2 diabetes, with the agonist increasing the expression of both co-activators and stimulating β-oxidation of fatty acids in the skeletal muscle of obese individuals [17].

In addition to their roles in regulating substrate metabolism in the heart, the PGC-1 co-activators also play important roles in cardiac development [18] and the switch from fatty acid metabolism to glycolytic metabolism following sepsis [19]. PGC-1β has also been implicated in arrhythmogenesis, particularly during adrenergic stress, in part associated with the accumulation of lysophospholipids in the heart [20]. 

In this study we have investigated the metabolic and lipidomic changes induced by high fat feeding in the PGC-1β knock out mouse [21] to investigate the role this co-receptor plays in regulating fat and carbohydrate metabolism in the heart. While GC-MS detected only a modest change in the length and degree of unsaturation of the total fatty acids in the heart, LC-MS based lipidomics demonstrated a major shift in intact lipids, with a decrease in a number of polar lipids and an increase in triglycerides.

**Figure 1:**
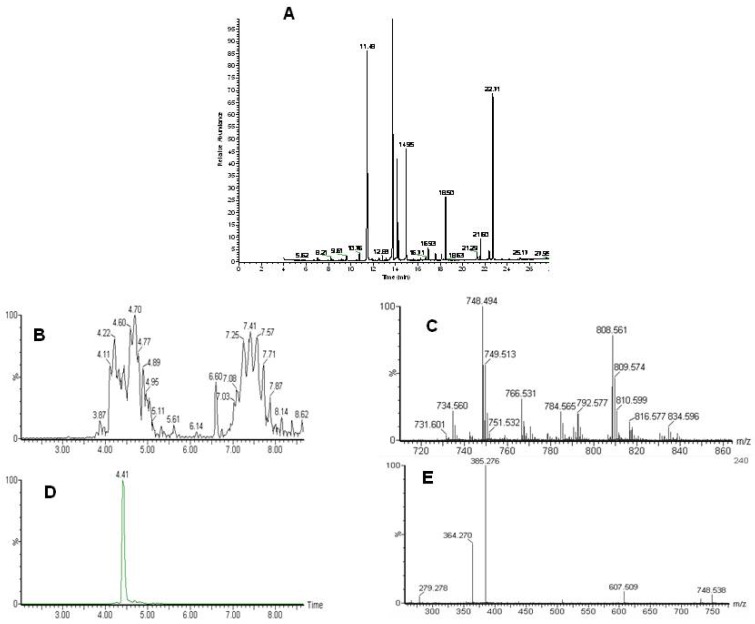
Analysis of the lipidome by GC-MS and LC-MS. A) A typical GC-MS chromatogram of fatty acid methyl esters in the organic fraction. Typical LC-MS data with identification. B) Total ion chromatogram of the LC-MS analysis of intact lipids. Phospholipids elute between 3.5 and 5.5 minutes and triacylglycerols between 6.5 and 8.5 minutes. C) Single scan mass spectrum from the LC-MS at 4.41 minutes D) extracted ion chromatogram for 748.5 E) MS-MS of 748.5 at 4.4 minutes. Characteristic fragments for a phosphoethanolamine with two fatty acids 22:0 and 22:6.

## 2. Results and Discussion

**Figure 2:**
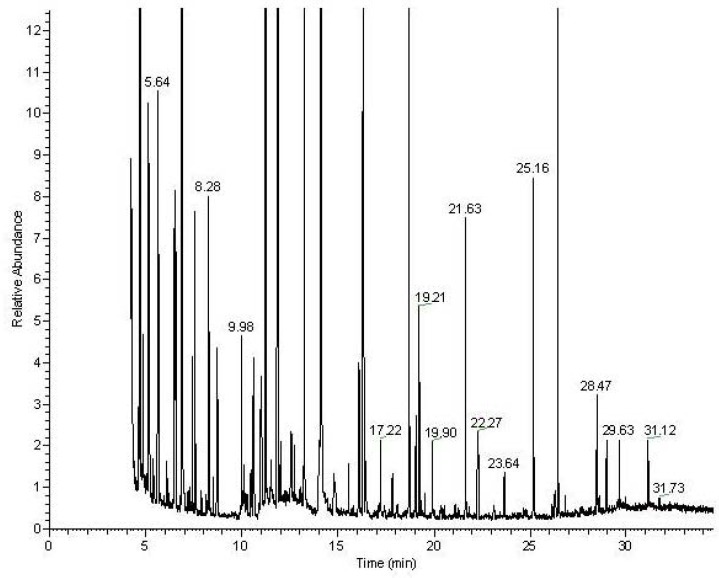
Typical GC-MS chromatogram of the MSTFA derivatized metabolites from the aqueous fraction of the heart tissue.

Following the chloroform/methanol lipid extraction of the heart tissues the organic, lipid containing fraction was analyzed both for total fatty acids by GC-MS and intact lipids by LC-MS (Figure 1). The aqueous fraction, without the lipids, was analyzed by NMR spectroscopy and GC-MS after appropriate derivatization (Figure 2). Lipid identification was performed by targeting the variables with a specific retention time and mass for MS-MS as demonstrated in Figure 1.

Table 1 summarizes the number of animals in each group and their weights. Only the male null mice were significantly lighter than their wildtype controls. The weight difference associated with genotype for male mice was not due to different amounts of food intake as this was monitored in the present study (data not shown) and has been observed previously in this genotype. However, there was no difference in the mass of the heart associated with genotype for either male or female animals (Student’s t-test; data not shown). 

**Table 1:** Summary of animals in each group used in this study with their average weights.

	Number of mice	Weight (SD) [g]	T-test
Male KO	10	34(4.2)	p=0.017
Male WT	8	40.2(5.2)	
Female KO	8	25.3(3.8)	p=0.16
Female WT	5	30(5.8)	

**Figure 3:**
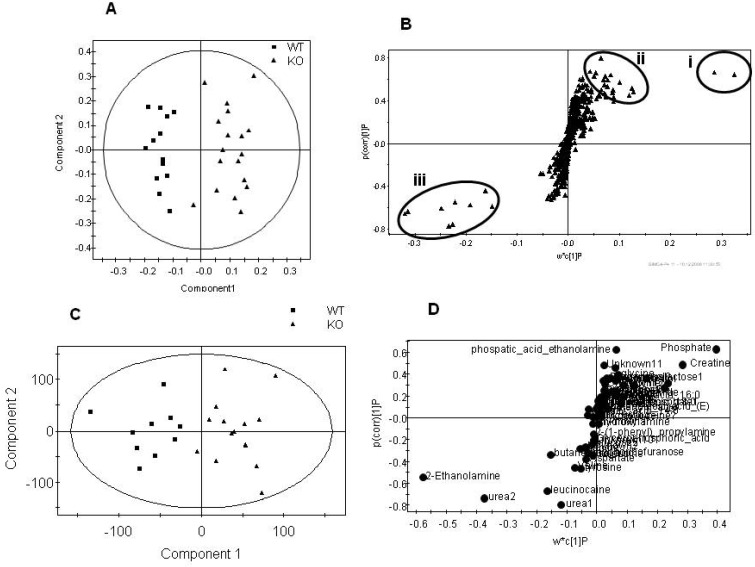
Summary plots for the multivariate analysis of the aqueous fraction for the total dataset comparing genotype, regardless of gender. A) Scores plot of the NMR spectra comparing tissue from the PGC-1β null mice compared with wild type controls (Q^2^= 79%). B) S-plot where the loading is plotted against the correlation between the variable and the score for the NMR spectral dataset. Signals in (i) are due to creatine (ii) are due to phospohcholine, glutamic acid, orotic acid, proline and ATP peaks in (iii) are due to taurine, alanine and succinate. C) Scores plot of GC-MS analysis of the aqueous fraction comparing tissue from the PGC-1β null mice and. wild type controls (Q^2^= 62%). D) S-plot where the loading is plotted against the correlation between the variable and the score for the GC-MS dataset.

**Figure 4:**
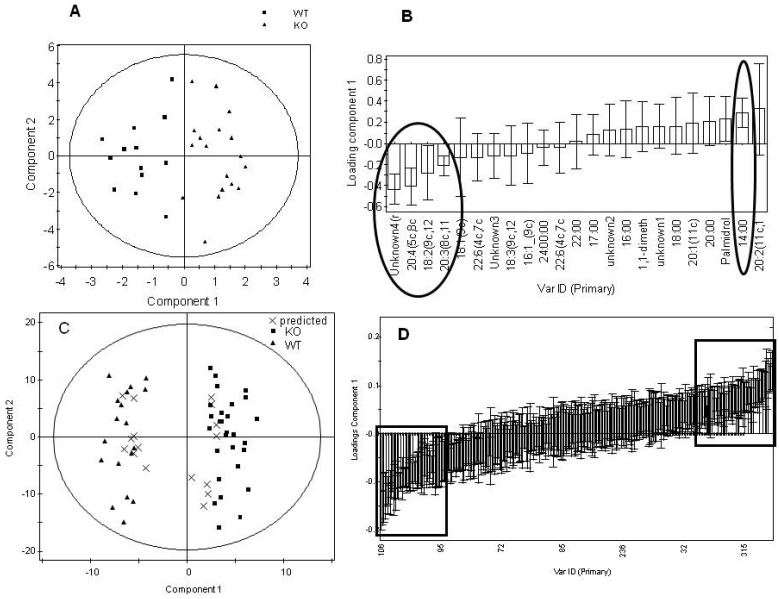
Summary plots for the multivariate analysis of the lipid fraction for the total dataset comparing genotype, regardless of gender. Analysis of organic GC-MS data. A) Scores plot of the GC-MS analysis of the lipid fraction to measure total fatty acid content in tissue from the PGC-1β null mice compared with wild type controls (Q^2^= 48%). B) Loadings plot sorted by loading value for the model in (A). The variables marked by the ovals were relevant to the model. Error bars represent 95% confidence interval as determined by jack-knifing. Analysis of LC-MS data. C) Scores plot comparing the LC-MS analysis of intact lipids with two injections for each sample comparing tissue from the PGC-1β null mice and the wild type controls (Q^2^= 89%). Two samples of each male KO, male WT, female KO and female WT were predicted correctly as shown in the plot. D) Loadings plot sorted by loadings value for the model in (D). The variables marked by the boxes were relevant to the model and identified. Error bars represent 95% confidence interval as determined by jack-knifing.

Multivariate statistics was used to analyze the NMR spectra and mass spectrometry chromatograms produced by the metabolomic/lipidomic analyses (Figure 3, Figure 4). Table 2 summarizes the PLS-DA models for all four analytical techniques. Using the Q^2^ score as a relative measure as to the robustness of the multivariate models, the metabolomic data from NMR spectroscopy, GC-MS and LC-MS confirms that the male mice were affected more by the gene deletion than the females. In addition models were built to investigate the metabolic differences purely associated with gender.

Considering the models built to discriminate gender first, there were no detectable differences for the aqueous fraction between genders, regardless of genotype, once the metabolomic data was corrected for mass by global normalization. However, the lipid fraction showed clear discrimination following analysis by both GC-MS for total fatty acids and LC-MS for intact lipids. However, as these changes were completely orthogonal to the subsequent genotype differences associated with the mutation they were not examined further. Indeed, where models could be built common metabolic changes were detected in the males, females and combined males and females groups. These metabolic changes are summarized in Table 3 and Table 4.

**Table 2:** Summary of the predictive capability of the models (Q^2^ values) for each of the techniques used to measure the metabolome in the heart.

	KO *vs*. WT; Both genders %	KO *vs*. WT male	KO *vs* WT female	female *vs* male
NMR	78	70	57	(no model)
GC-MS (Aq)	61	79	(no model)	(no model)
GC-MS (Org)	48	32	(no model)	80
LC-MS	89	80	89	91

**Table 3:** Changes in the concentration of metabolites detected by NMR spectroscopy and GC-MS using the combined male and female dataset. Similar changes were detected in the individual gender models.

	Increased in Knock Out	Decreased in Knock Out
NMR	creatine	Taurine
	ATP	alanine
	proline	succinate
	oratate	
	glutamine	
	glutamate	
	phosophcholine	
GC-MS (aq.)	Phosphate	Urea
	Glutamate	valine
	creatine	leucine
	Glutamine	2-ethanolamine
		Tyrosine
GC-MS (org.)	14:00	Unknown (long chain polyunsat.)
	(palmidrol, 20:0)	20:4n-6
		20:3n-6
		18:2n-6

**Table 4:** Changes in metabolite levels as detected by LC-MS in the combined gender model. Assignments were confirmed by MS/MS. Where individual fatty acids are not specified it was not possible to definitively identify the lipid beyond head group, total chain length and degree of unsaturation. Unknowns 1 and 2 are hydrophobic molecules with a retention time of 7.41 and 6.75 minutes and mass (M+NH_4_^+^) of 880.65 and 812.6; both of these unknowns have a fragment ion of 197 Da. Similar changes were detected in the individual gender models. Note it cannot be determined with certainty which fatty acids are in a given position from the fragmentation patterns.

	Increased in Knock out	Decreased in Knock Out
LC-MS	PC(16:0/22:6)	PC(18:2/22:6)
	PC(18:0/22:6)	PC(40:7) 2 isomers
	PE(22:6/20:4)	PC(36:4)
	PE(40:6)	PC(38:5)
	PE(22:0/22:6)	PC(36:3)
	PE(22:6/17:1) or PE(38:0)	PC(34:1)
	TAG(18:2/18:2/22:6)	PC(40:5)
	TAG(55:2)	PC(38:4)
	TAG(18:1/18:2/22:6)	PC(18:0/22:4)
	TAG(20:3/18:2/18:2)	PE(40:7)
	TAG(20:5/18:2/18:1)	PE(36:2)
	TAG(18:2/20:3/16:0)	PE(20:4/18:0)
	TAG(58:4)	PE(40:5)
	TAG(18:1/18:1/18:2)	PE(16:0/22:6)
	TAG(56:7)	Unknown 1
	TAG(58:3)	Unknown 2
	TAG(18:1/18:2/17:1)	
	TAG(54:3) mixture containing (16:0/18:1/18:2/18:0/20:1/20:2/20:3)	
	TAG(18:1/18:2/18:0)	
	TAG(18:1/18:1/20:1)	

The largest change in the aqueous fraction as measured by both NMR spectroscopy and GC-MS was associated with an increase in creatine in the heart tissue from the knock-out mice. Phosphocreatine acts as a store of phosphorylation potential in muscle, buffering the use of ATP. PGC-1β plays an important role in mitochondrial biogenesis [14,15], and hence the energetics of the cell. However, the function of PGC-1β can be compensated for by an upregulation of PGC-1α, and in the present study ATP was found to be increased in the heart tissue of the mutant, and the increased concentration of creatine may be a further compensatory mechanism for a failure to express PGC-1β. In mice where either mitochondrial or cytosolic or both isoforms of creatine kinase are deleted, and there is a marked reduction in phosphocreatine, the deletion is compensated by a marked increase in mitochondrial number within muscle cells [25]. In the PGC-1β knock-out mouse, the increased creatine may represent an increased buffering capacity of the cardiac muscle tissue to compensate the loss of PGC-1β and its role in mitochondrial biogenesis and function. Furthermore, the detected increase in glutamate, which is readily converted into 2-oxoglutarate via the malate/aspartate shuttle, and the decrease in succinate suggests altered mitochondrial metabolism in the knock-out animal. There was also a decreased concentration of taurine in the heart, with taurine playing an important role in buffering cardiac calcium. Reductions in taurine have been correlated with arrhythmia in the heart [26,27], and we have previously reported that the PGC-1β null mouse is prone to certain types of arrhythmogenesis [20].

While the changes in the aqueous fraction were relatively modest, the failure to express PGC-1β had a profound effect on the lipidome of the heart tissue. In terms of the total fatty acid changes there was a profound decrease in long chain polyunsaturated fatty acids, and a relative increase in the short chain fatty acid myristic acid. Even more profound changes were detected in the intact lipids with a marked increase in triglycerides detected in the heart tissue of the PGC-1β knock-out mouse. The increased accumulation of triglycerides in the heart suggests that either the heart of the PGC-1β knock-out mouse has an increased reliance on fatty acid oxidation or there was increased accumulation of triglycerides in the heart because these lipids were not oxidized in other tissues following the high fat feeding used in the present study. However, countering the second hypothesis is the reduced animal weight of the male knock-out mice compared with their wild type controls, suggesting that the males may be resistant to diet induced weight increases presumably as a result of increased capacity for fatty acid oxidation. We have previously reported a slower increase in mass for the PGC-1β knock-out mice when fed on a chow diet [21]. While the resistance to increased body mass on a high fat diet was not reported in another previous study [28] this separately generated PGC-1β null mouse line did have a trend for decreased body mass following 80 days of high fat feeding which may have become statistically significant if extended to the period used in the present study. Furthermore, the global increase in myristic acid compared with a decrease in long chain polyunsaturated fats in the heart tissue from the mutant also suggests increased β-oxidation which would increase the relative proportion of short chain fatty acids. Given the expression patterns of the PPARs and their co-activators, the increased β-oxidation is most likely across skeletal muscle and the liver, in addition to an increased capacity in the heart. Thus, overall it would appear that heart tissue from the PGC-1β knock-out mouse has increased capacity for triglyceride metabolism despite reported reductions in mitochondrial size and key transcripts associated with the citric acid cycle, the electron transport chain and oxidative phosphorylation [21,28].

The hypothesized increase in β-oxidation of triglycerides in the heart of PGC-1β knock-out mice may seem counter intuitive given that PGC-1β has a role in regulating the expression of carnitine palmitoyltransferase I, as well as regulating mitochondrial function. However, one caveat with any functional genomic study relying on gene deletions to induce a phenotype is that the ultimate phenotype measured in adulthood represents the effects of development without the deleted gene and any adaptations that occur in response to this. PGC-1α shares many of the functions of PGC-1β, and in brown adipocytes where either are deleted the cell lines show mild phenotypes, particularly when compared to the double mutant cell lines which have severely impaired mitochondrial biogenesis [15]. Thus, the increased β-oxidation may arise from increased expression of PGC-1α, as has been observed previously in a variety of tissues in the PGC-1β null mouse including skeletal muscle [28], to counteract the loss of PGC-1β. This effect appears to extend to global systemic metabolism in the male PGC-1β mice where we have shown a significant decrease in weight compared with their wild type controls following the high fat feeding used in the present study. However, an increased expression of PGC-1α cannot explain the phenotype completely as a number of transcripts involved in mitochondrial metabolism are still decreased in the heart following both chow and high fat feeding [21,28]. To address the role PGC-1β plays in regulating heart metabolism it may be more beneficial to generate heart–specific inducible PGC-1β null mice in order to circumvent the upregulation of other genes to counter the loss of PGC-1β in the heart and avoid the complications associated with altered systemic metabolism associated with global knock-outs. 

One of the most profound metabolic changes detected in the heart tissue from PGC-1β null mice was the increase in specific triglycerides compared with tissue from the wildtype controls, with 14 triglycerides being detected as being increased and none as decreased. Furthermore, the LC-MS data examining the intact lipid changes in the heart produced much more discriminatory changes than that of the GC-MS analysis of total fatty acids demonstrating the importance of being able to determine the head group that particular fatty acids are associated with. This increased level of metabolite identification appears to be the more likely explanation for the increase in discrimination rather than differences in sensitivity, as the GC-MS analysis of total fatty acids is a fairly routine and robust assay. The increase in triglycerides in the heart tissue appears somewhat counterintuitive with the decrease in mass of the whole animal following high fat feeding and the detection of increased short chain fatty acids as detected by GC-MS. However, if the PGC-1β null mice do demonstrate increased capacity for β-oxidation the increased TAGs found in the heart tissue may represent increased TAG stores. Furthermore, Sonoda and co-workers [28] reported increased accumulation of triglycerides in the liver of PGC-1β null mice following high fat feeding despite no increase in body mass or adiposity in these animals. They hypothesized that this accumulation arose because PGC-1β has a role in regulating the synthesis and storage of fatty acids in the liver. Thus, the cardiac accumulation of triglycerides may represent a pathological accumulation in peripheral tissues. However, one of the problems with our approach is that by measuring total concentrations of given metabolites it is difficult to judge changes in flux of specific pathways, and thus, a stable isotope approach may be necessary to determine how increased triglycerides within the heart may result from increased demand by β-oxidation or a failure to regulate triglyceride uptake in the heart.

While we focused on the results from the combined dataset, a consistent result across all the analytical platforms was that the differences were larger in the male dataset compared with the female dataset, particularly when comparing the Q^2^ values for the individual PLS-DA models. This may partly reflect the lower replicate number used in the female dataset. However, PGC-1α and PGC-1β both interact with the estrogen related receptors (ERRs), particularly in the heart between PGC-1α and ERR-α [29]. The ERRs are nuclear hormone receptors which are closely related to the estrogen receptor and interact with estrogen. They regulate the transcription of genes involved in a wide variety of processes including the key metabolic processes of mitochondrial biogenesis, oxidative phosphorylation, gluconeogeneis, and fatty acid metabolism [30,31,32,33,34]. Thus, some of the differences between the two genders may arise from interactions between PGC-1α and ERR-α to counteract the loss of PGC-1β. 

## 3. Experimental Section

### 3.1. Animal Handling

PGC-1β null mice were generated using a triple LoxP targeting vector as described previously [21]. In brief, the targeting vector containing a floxed neomycin phosphotransferase selectable marker cassette inserted into intron 3 and a single LoxP site inserted into intron 5, resulting in deletion of exons 4 and 5. Heterozygous triple LoxP mice crossed with ROSA26-Cre mice generated heterozygous PGC-1β mice, which were further bred to generate homozygous PGC-1β mice. Genotypes were confirmed by Southern blotting. Animal protocols and procedures used in this study were approved by the UK Home Office [UK Animals (Scientific Procedures) Act 1986], and the Cambridge University ethics review committee. Animals were fed a high fat diet for three months post weaning and killed at 4 months of age. The heart was rapidly removed (< 30 s post mortem) and then frozen in liquid nitrogen and stored at -80 ºC prior to analysis.

### 3.2. Metabolite Extraction

Metabolites were extracted from left ventricle tissue using a chloroform-methanol procedure adapted from the method of Bligh and Dyer [22]. In brief, ~100 mg of heart tissue was pulverized in 600 µl chloroform: methanol (1:2) using 25 Hz setting for two minutes in a Qiagen TissueLyser (Retsch, Haan Germany). Two hundred microliters of chloroform and then 200 µl deionized water (HPLC grade, Fisher Scientific, Leicestershire UK) was added to each sample. Samples were vortexed briefly, then centrifuged for 20 minutes at 10,000g. A 50 µl portion of the aqueous layer and a 100 µl portion of the organic layer were removed for each analysis described below and dried down separately. Aqueous samples were dried overnight, without heating, in an evacuated centrifuge (Eppendorf, Hamburg, Germany). Organic samples were dried overnight in a fume hood under a stream of nitrogen gas. All extracted samples were stored at −20ºC until required.

### 3.3. Gas Chromatography-Mass Spectrometry of the Organic Fraction

Samples were derivatized by acid catalysed esterification [23]. Briefly, 150 µl chloroform: methanol (1:1) and 100 µl of 10% BF_3_ (Sigma-Aldrich) in methanol was added to each of the organic samples set aside for GC-MS analysis. Samples were briefly vortexed and heated for 90 minutes at 80 ºC. Once samples had cooled, 0.6 ml hexane (GC analysis grade, Sigma-Aldrich) and 0.3 ml water (HPLC grade, Fisher Scientific, Leicestershire UK) was added. After vortexing, the organic layer (upper layer) was transferred to vials and left to dry overnight. Samples were reconstituted with 0.5 ml hexane and injected into a Finnigan GC Ultra DSQ II (Thermo Scientific, Massachusetts, USA) using a 30m Thermo TR-FAME column and a 1 in 25 split. The injection volume was 3 µl. The initial column temperature was 60 ºC for 2 minutes. This was then increased by 15 ºC/min to 150 ºC, and then increased by 4 °C/min to a final temperature of 230 ºC. Each sample was run for 28 minutes and data was collected across a mass to charge ratio range of 50-650 m/z with a starting delay of 4 minutes.

### 3.4. Gas Chromatography- Mass Spectrometry Analysis of The Aqueous Fraction

Samples were derivitized based on the protocol by Gullberg and co-workers [24]. Briefly, to each dried sample 30 µl of 20 mg/ml methoxylamine hydrochloride (98%, Sigma-Aldrich) in pyridine (Rathburn chemicals, Walkerburn, UK) was added. Each sample was vortexed briefly and left to stand at room temperature for 17 hours. After the addition of 30 µL of N-Methyl-N-(trimethylsilyl)trifluoroacetamide (MSTFA) to each sample, they were left to stand at room temperature for one hour. The samples were diluted 1:10 in hexane and run on a Finnigan Trace GC Ultra DSQ II (Thermo Scientific, Massachusetts, USA) containing a 30m Zebron Capillary GC Column (Phenomenex, California USA). A volume of 2 µl of sample was injected and samples were run splitless. The initial column temperature was 70 ºC. This was increased by 10 ºC/ min to 130 ºC, and then increased by 5 °C/min to a final temperature of 310 ºC, where it was held for 5 minutes. Each sample was run for 35 min and data was collected across a mass to charge ratio range of 50-650 m/z after a starting delay of 4 min.

### 3.5. Data Analysis of GC-MS Chromatograms

GC-MS data was analyzed using QuanBrowser in XCalibur (version 2.0, Thermo Scientific, Massachusetts USA). A method was written to integrate extracted ion chromatographic peaks that were identified using the National Institute of Science and Technology (NIST) library. Peak integrals were normalised and exported for further analysis.

### 3.6. ^1^H-NMR Spectroscopy of Aqueous Fraction

The dried extracts of the aqueous phase were rehydrated in 600 µl of D_2_O, buffered with 200 mM Na_2_HPO_4_, 40 mM Na_2_H_2_PO_4_.2H_2_O, 0.1% NaN_3_, and 1mM sodium-3-(tri-methylsilyl)-2,2,3,3-tetradeuteriopropionate (TSP, Cambridge Isotope Laboratories, Massachusetts USA) dissolved in D_2_O. Spectra were acquired using a 5 mm inverse-detection TXI probe in an 11.7 Tesla magnet (500.3 MHz proton frequency) interfaced to a Bruker AVANCE II+ spectrometer (Bruker, Coventry, UK). Spectra were acquired at 30°C and were the sum of 64 transients acquired into 32K data points using the noesypr1d pulse sequence with the following parameters: Relaxation Delay (RD)-90°-t_1_-90°-t_m_-90°-acquire; RD = 1.5s, t_1_ = 4 s; TR = 2s. The sweep width was 8000 Hz and the water resonance was suppressed by low power irradiation during the mixing time (t_m_) of 50ms and the relaxation delay. Spectra were Fourier transformed following multiplication by a 0.3 Hz exponential function.

Spectra were processed (phased and baseline corrected) using the ADC/Spec Manager (Version 9.15) software. The phase and the baseline were manually corrected. Integration of peaks was processed for regions 0.22–4.67 ppm and 5.23–9.36 ppm, excluding the water resonance. Intelligent bucketing with an approximate width of 0.01 ppm was used, producing 599 integral buckets in total. The raw integrals were normalised to total intensity and analysed further by multivariate statistics.

### 3.7. LC-MS of Intact Lipids

Lipid extracts were re-dissolved in 600 µl 2:1 methanol/chloroform and diluted 10 fold before 4 µl were injected into a UPLC Acquity™ (Waters, Milford, USA) with a C8 column (ACQUITY UPLC™ BEH C8 2.1 × 100 mm, 1.7 µm, Waters). Mobile phase A was made up of water with 0.1% formic acid and 10 mM ammonium acetate, while phase B was acetonitrile:isopropanol (5:2) with 0.1% formic acid and 10 mM ammonium acetate. The analytes were eluted from the column over 10 minutes with a gradient of 60 to 100% B followed by 2 minutes at 100% B and 2 minutes of column re-equilibration at the starting condition 60% B. The column flow rate was 600 μl/ min. The UPLC was coupled to a Q-ToF micro mass spectrometer (Waters, Milford, USA) with a 1:1 eluent split. After every 15 sample injections a pooled sample followed by a blank were injected in order to ensure consistent performance of the system. The randomized sample list of extracts was run twice. MassLynx (Waters, Milford, USA) was used to identify peaks by retention time and mass. De-isotoping was turned off and the thresholds were set low to allow MassLynx to detect over 7000 peaks. In a second step all variables with missing values in more than 10 spectra were discarded along with all signals of low intensity in the pooled sample spectra (< 0.05% parts of the sum of all variables) as relative quantification becomes unreliable with low intensity peaks. This left 324 variables that were normalized to total intensity of the 324 variables for every sample. The resulting data table was further analyzed with multivariate statistics as described below. The variables that were determined to be of interest were used to set up a targeted MS-MS method, recording a total of more than 40 targeted fragment spectra in order identify the altered metabolites.

### 3.8. Data Analysis by Multivariate Statistics

Data matrices from all four measurements described above were exported to SIMCA-P + 11 (Umetrix, Umea, Sweden) for multivariate analysis by Principal Components Analysis (PCA) and Partial Least Squares Discriminant Analysis (PLS-DA). Prior to analysis, Pareto scaling was used on the NMR and the GC-MS (Aq.) datasets and univariate on the LC-MS and GC-MS (Org.) datasets. PCA was used to remove outliers (6 samples for the aqueous fraction analysed by GC-MS and 3 samples for LC-MS). Various PLS-DA models used in the results section were built and validated using the goodness of fit score (Q^2^) and the validation tool within SIMCA whereby class membership is randomly permuted and the Q^2^’s for these random models compared with the true value. For validated models (negative Q^2^ intercept and negative R^2^ regression in the permutation test) Orthogonal Partial Least Squares-Discrimant analysis (OPLS-DA) was performed to establish which variables drive the separation.

## 4. Conclusions

In conclusion, we have used a combined metabolomic and lipidomic approach to study metabolic differences in the heart tissue of the PGC-1β null mouse. Profound metabolic changes were detected particularly in lipid metabolism, with the cardiac tissue of PGC-1β null mice having increased concentrations of specific triglycerides but reduced concentrations of long chain polyunsaturated fatty acids. The data also suggests gender specific differences in the metabolic changes induced by a failure to express PGC-1β, possibly through interactions between PGC-1α and ERR-α.
